# The use of successive systolic differences in photoplethysmographic (PPG) signals for respiratory rate estimation

**DOI:** 10.1016/j.heliyon.2024.e26036

**Published:** 2024-02-08

**Authors:** Erick Javier Argüello-Prada, Katherin Daniela Marcillo Ibarra, Kevin Leonardo Díaz Jiménez

**Affiliations:** Programa de Bioingeniería, Facultad de Ingeniería, Universidad Santiago de Cali, Cali-Colombia, Calle 5 # 62-00 Barrio Pampalinda, Santiago de Cali, Valle del Cauca, Colombia

**Keywords:** Respiratory rate, Photoplethysmography, Respiratory-induced variation, Successive systolic differences, Estimation error

## Abstract

Most PPG-based methods for extracting the respiratory rate (RR) rely on changes in the PPG signal's amplitude, baseline, or frequency. However, several other parameters may provide more valuable information for accurate RR computation. In this study, we explored the capabilities of the respiratory-induced variations in successive systolic differences (RISSDV) of PPG signals to estimate RR. We partitioned fifty-three publicly available recordings into eight 1-min segments and identified peaks and troughs of the PPG signals to quantify respiratory-induced variations in amplitude (RIAV), baseline (RIIV), frequency (RIFV), and peak-to-peak amplitude differences (RISSDV). RR values were extracted by determining the peak frequency of the power spectral density of the four variations and the reference respiratory signal. We assessed each feature's performance by computing the root-mean-squared (RMSE) and mean absolute errors (MAE). RISSDV errors were significantly lower than those of RIAV (RMSE and MAE: *p* < 0.001), RIIV (RMSE: *p* < 0.01; MAE *p* < 0.05), and RIFV (RMSE and MAE: *p* < 0.001), and it appeared less sensitive to absent or missed PPG pulses than respiratory-induced frequency variations. Further research is necessary to extrapolate these findings to subjects under ambulatory rather than stationary conditions, including pediatric and neonatal populations.

## 1 Introduction

Arguably, no other sensing modality for physiological monitoring has gained as much interest over the last decades as photoplethysmography (PPG). This technique employs a light source to illuminate the tissue and a photodetector to capture the blood flow-induced variations in reflected (or transmitted) light intensity. Thus, the wave-like motion of the blood through the vessels is converted into an electrical signal known as the PPG signal [1]. Given its non-invasive character, simplicity, and low cost, PPG has been widely proposed as an alternative to overcome the limitations associated with the most commonly used methods to monitor several physiological variables, including the respiratory rate (RR). RR represents the number of breaths per minute (denoted as *bpm*), and there is substantial evidence of its relevance in the early prediction of deterioration in the patient's condition [[2], [3], [4]]. Still, recent findings reveal a poor understanding of the importance of accurate monitoring and recording of RR [5,6].

As a result of the complex interactions taking place between the respiratory and cardiovascular systems, breathing is expected to have a significant influence on peripheral blood flow. The changes produced by the respiratory cycle in the amplitude, baseline, and frequency of the PPG signal are often referred to as RIAV (respiratory-induced amplitude variation), RIIV (respiratory-induced intensity variation), and RIFV (respiratory-induced frequency variation), respectively. These three parameters have been well-documented [[7], [8], [9]], and most algorithms developed for extracting RR from PPG signals are based on them. Whereas some methods use the information provided by only one of those features (RIAV: [10,11]; RIIV: [[12], [13], [14]]; RIFV: [15]), others combine them to compensate for the limitations of each one and reduce the error in estimating RR [[16], [17], [18], [19]]. However, results reported by some studies suggest that respiratory-induced variations other than RIAV, RIIV, and RIFV could be more valuable for accurate RR computation. For instance, Cernat and colleagues [20] found that amplitude differences between successive maxima of PPG signals (henceforth termed respiratory-induced variation in successive systolic differences - RISSDV) provide a normalized root-mean-squared error lower than those obtained by using RIAV, RIIV, and RIFV. On the other hand, the authors used only seven 8-min recordings collected from children (1–8 years old), thereby limiting the generalization of the findings. Furthermore, whether or not the combined use of RISSDV and other respiratory-induced variations could decrease the error and enhance the accuracy of RR estimates was not reported. Therefore, the present study aims to perform a more extensive assessment of the value of RISSDV in extracting RR from PPG signals. Unlike previous research conducted in this regard [20], this work considers a larger dataset and several other performance assessment metrics, such as the mean absolute error (MAE) and Bland-Altman plots. To investigate whether RISSDV provides a more accurate estimation of RR than that of the most commonly referred RIVs (i.e., RIAV, RIIV, and RIFV) could contribute to developing more efficient methods for computing RR from PPG signals.

## 2 Methods

### 2.1 Database

We used a publicly available dataset containing 8-min recordings from 53 critically ill patients at the Beth Israel Deaconess Medical Center (BIDMC), Boston, USA, with ages ranging from 19 to 90+ years (median age: 64.81; 32 females) [18,21]. Each recording includes PPG and thoracic impedance respiratory signals sampled at 125 Hz. We considered the respiratory signal as the reference for all comparisons.

#### 2.1.1 Signal preprocessing

We imported 53 recordings from the BIDMC database to the MATLAB programming environment (R2017a; The MathWorks Inc. Natick, USA). PPG and respiratory signals were band-pass filtered to remove the DC component and high-frequency noise (PPG: 0.05–10 Hz; respiration: 0.05–1 Hz). We did not detrend PPG signals as, like in several other studies [10,18,19], we wanted to preserve the respiratory-induced baseline variation (i.e., RIIV).

#### 2.1.2 Respiratory rate (RR) computation

**Fig. 1:**
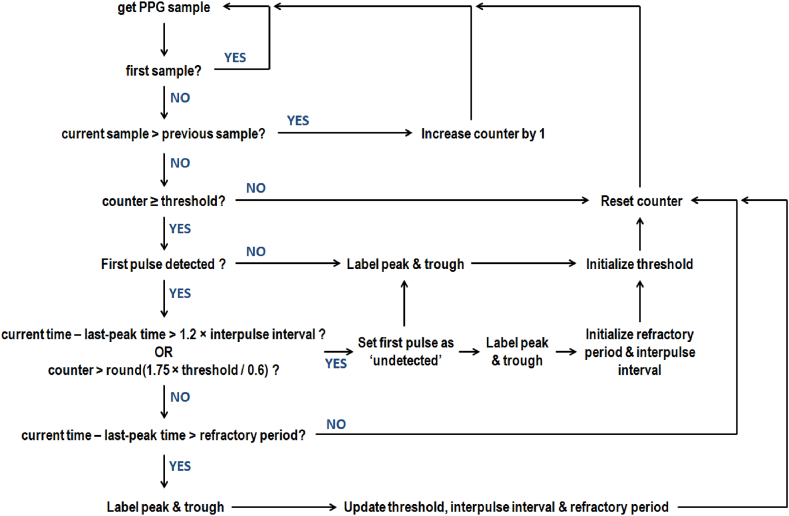
Pseudocode of the peak-trough detection method.

After signal preprocessing, we partitioned each recording into eight non-overlapping 1-min segments. Information regarding the amplitude and temporal location of PPG peaks and troughs to compute RIVs was acquired using an adaptation of the method introduced by Argüello-Prada [22]. On the one hand, we preserved the overall approach, which counts the times the amplitude of the current sample is higher than that of the previous one to detect a systolic upslope. Each pulse peak and through are identified if the counter reaches or exceeds a certain threshold. However, if the counter remains below the threshold value, that upslope is ignored, and the counter resets. On the other hand, we added several modifications to avoid overdetection and missed peaks. After detecting the first pulse, further pulses are labeled if the time difference between successive peaks (i.e., the interpulse interval) is higher than a refractory period, which is constantly updated depending on how much time has elapsed since the last detected peak. If the counter exceeds 1.75 times the updated threshold, detection is restarted, so the next PPG pulse is treated as the first (see Fig. 1).

RIAV, RIIV, RIFV, and RISSDV were computed as the trough-to-peak amplitudes of each PPG pulse, the absolute peak amplitudes of each PPG pulse, the time intervals between successive systolic peaks, and the amplitude differences between successive systolic peaks, respectively. We removed non-respiratory frequencies by applying an FIR band-pass (0.05–0.6 Hz) filter, as done by Selvakumar and coworkers [10]. For each 1-min segment, five RR values were extracted by identifying the peak frequency of the power spectral density (PSD) of the respiratory signal (reference value) and the four RVIs (estimated values). This task was achieved using the MATLAB "max" function, which returns the index of the PSD maximum value (and, therefore, the corresponding frequency). To compute the PSD, we considered the utilization of the fast Fourier transform (FFT), as it has been the most widely used method among the abovementioned studies [10,15,17,19,23]. Given that the FFT approach requires evenly sampled data for PSD estimation, it is necessary to interpolate and resample RIVs. However, interpolating and resampling unevenly spaced data can produce a low-pass filtering effect at upper-frequency bands, thereby interfering with power spectrum estimation [24]. In this regard, several authors have found that the Lomb-Scargle (LS) periodogram is more suitable than FFT for PSD estimation of unevenly sampled data [24,25]. Therefore, we used the LS method to compute the PSD of the four RVIs and the respiratory signal over each 1-min segment. The resulting power spectrograms were plotted over the frequency range from 0.067 to 0.5 Hz (i.e., 4 to 30 bpm) to account for RR values beyond the frequency band commonly attributed to respiratory sinus arrhythmia in heart rate variability (HRV) analysis (0.15–0.4 Hz) [26].

Regarding whether or not the use of RISSDV in combination with other respiratory-induced variations can decrease the error of RR estimates, we used the 'fusion' method proposed by Karlen and colleagues [19] to combine three of the four original estimations. Thus, four additional RR estimates were obtained as follows.

• RR_AIF_: (RR_RIAV_ + RR_RIIV_ + RR_RIFV_)/3
• RR_SIF_: (RR_RISSDV_ + RR_RIIV_ + RR_RIFV_)/3
• RR_ASF_: (RR_RIAV_ + RR_RISSDV_ + RR_RIFV_)/3
• RR_AIS_: (RR_RIAV_ + RR_RIIV_ + RR_RISSDV_)/3

#### 2.1.3 Performance assessment and statistical analysis

The estimated RR values obtained from the four RIVs and the four combinations (see above) were compared with the RR computed from the reference respiratory signal. We assessed the performance of each feature by calculating the root-mean-squared error (RMSE) and the mean absolute error (MAE) in bpm, defined by Equations (1), (2):

(1) $$RMSE=\sqrt{\frac{1}{N}\sum_{i=1}^{N}{\left({RR}_{est}\left(i\right)-{RR}_{ref}\left(i\right)\right)}^{2}}\text{,}$$

(2) $$MAE=\frac{1}{N}\sum_{i=1}^{N}\left|{RR}_{est}\left(i\right)-{RR}_{ref}\left(i\right)\right|\text{,}$$

where *RR*_*est*_ and *RR*_*ref*_ denote the estimated and reference RR values, respectively, and N represents the total of observations. A Kruskal-Wallis test followed by pairwise Mann-Whitney tests with Bonferroni correction was conducted on the RMSE and MAE of all RIV-based RR estimations using the R statistical software [27] (we considered a *p*-value <0.05 as statistically significant). We also computed the classical Bland–Altman plot with mean difference's 95% confidence intervals [28] to evaluate the agreement and interchangeability between PPG-derived estimations and actual RR values.

## 3 Results

**Fig. 2:**
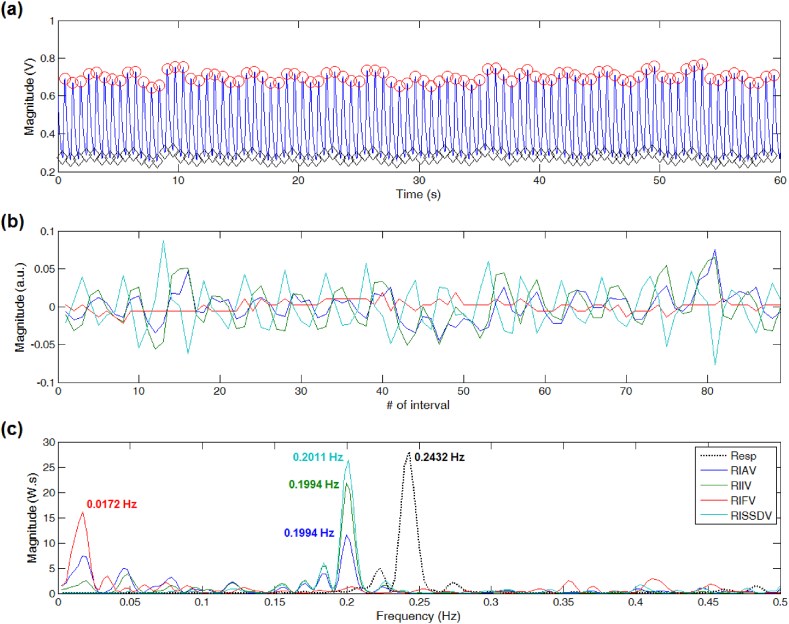
Respiratory rate (RR) estimation on a good-quality PPG signal. (a) Results of peak-trough detection. (b) PPG-derived respiratory-induced variations. (c) RR extraction from computed power spectral density via Lomb-Scargle periodogram.

**Fig. 3:**
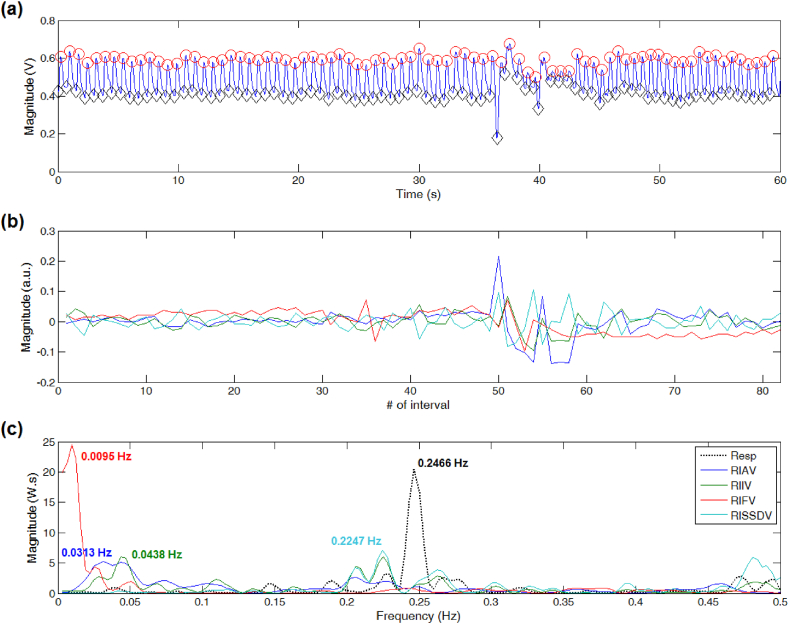
Respiratory rate (RR) estimation on a PPG signal showing sudden but slight contour changes. (a) Despite the signal's amplitude and baseline fluctuations, the peak-trough detection process was continuous. (b) Some respiratory-induced variations become irregular due to unexpected changes in the PPG waveform. (c) Comparison between PPG-derived RR estimates and the reference peak frequency.

**Fig. 4:**
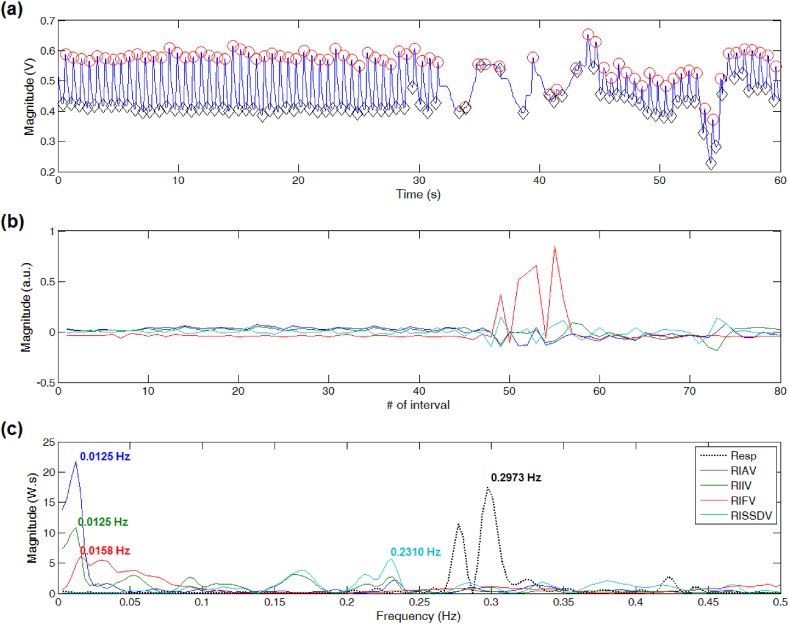
Respiratory rate (RR) estimation on a PPG signal showing severe contour changes. (a) Missed or false detections due to notorious fluctuations in signal amplitude and baseline. (b) Respiratory-induced frequency variations are highly sensitive to long-lasting discontinuities in the peak-trough detection process. (c) RR estimation errors.

For PPG signals showing little differences between neighboring peaks and troughs' locations, the dominant frequency of DEPs derived from RISSDV, RIIV, and RIAV approximated the reference peak frequency value (see Fig. 2). However, only estimations derived from RISSDV and RIIV were comparable to the reference value when PPG signals showed sudden or unexpected contour changes, even if the peak-trough detection process was continuous, as shown in Fig. 3. For PPG segments showing severe disturbances producing long-lasting discontinuities in the peak-trough detection process (i.e., several missed or false detections), only the peak frequency of the RISSDV-derived DEP approximated the reference value (see Fig. 4).

**Fig. 5:**
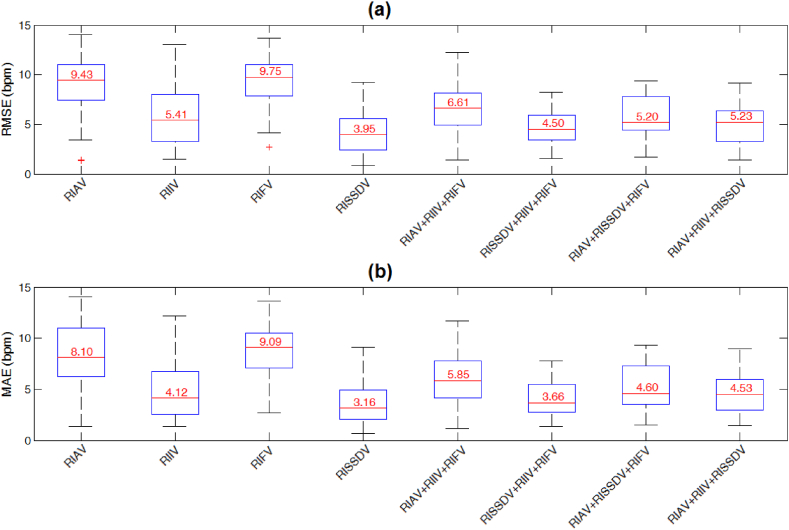
Distribution of (a) the root-mean-squared error (RMSE) and (b) the mean absolute error (MAE) for all respiratory-induced variation-based RR estimations.

Fig. 5 shows the median values of RMSE and MAE of the eight RIV-based RR estimations. The Kruskal-Wallis test revealed significant differences between errors (RMSE: χ^2^(7) = 146.04, *p* < 0.001; MAE: χ^2^(7) = 132.36, *p* < 0.001). As shown in Table 1, Table 2, post hoc multicomparison tests showed that RMSE and MAE values of RSSIDV were significantly lower than those of RIAV (*p* < 0.001 for both errors), RIIV (*p* < 0.01 and <0.05), RIFV (*p* < 0.001 for both errors), RIAV + RIIV + RIFV (*p* < 0.001 for both errors), and RIAV + RISSDV + RIFV (*p* < 0.01 for both errors). Moreover, there was no significant difference between RMSEs of RISSDV and RIAV + RIIV + RISSDV (*p* = 0.125), albeit MAEs were significantly different (*p* < 0.05). Statistically comparable errors between RSSIDV and RISSDV + RIIV + RIFV were observed (*p* = 0.337 and 0.186).

**Table 1:** Statistical comparison of the root-mean-squared errors of all RIV-based RR estimations. Asterisks denote significant differences between computed errors (Kruskal-Wallis test and post hoc pairwise Mann-Whitney tests with Bonferroni correction, *p < 0.05, **p < 0.01, ***p < 0.001).

	RIAV	RIIV	RIFV	RISSDV	RIAV + RIIV + RIFV	RISSDV + RIIV + RIFV	RIAV + RISSDV + RIFV	RIAV + RIIV + RISSDV
RIAV	–	***	0.482	***	***	***	***	***
RIIV	***	–	***	**	0.087	0.068	0.966	0.21
RIFV	0.482	***	–	***	***	***	***	***
**RISSDV**	***	**	***	–	***	0.337	**	0.125
RIAV + RIIV + RIFV	***	0.087	***	***	–	***	0.079	**
RISSDV + RIIV + RIFV	***	0.068	***	0.337	***	–	0.074	0.566
RIAV + RISSDV + RIFV	***	0.966	***	**	0.079	0.074	–	0.226
RIAV + RIIV + RISSDV	***	0.21	***	0.125	**	0.566	0.226	–

**Table 2:** Statistical comparison of the mean absolute errors of all RIV-based RR estimations. Asterisks denote significant differences between computed errors (Kruskal-Wallis test and post hoc pairwise Mann-Whitney tests with Bonferroni correction, *p < 0.05, **p < 0.01, ***p < 0.001).

	RIAV	RIIV	RIFV	RISSDV	RIAV + RIIV + RIFV	RISSDV + RIIV + RIFV	RIAV + RISSDV + RIFV	RIAV + RIIV + RISSDV
RIAV	–	***	0.407	***	***	***	***	***
RIIV	***	–	***	*	*	0.333	0.442	0.811
RIFV	0.407	***	–	***	***	***	***	***
**RISSDV**	***	*	***	–	***	0.186	**	*
RIAV + RIIV + RIFV	***	*	***	***	–	***	0.109	**
RISSDV + RIIV + RIFV	***	0.333	***	0.186	***	–	0.083	0.466
RIAV + RISSDV + RIFV	***	0.442	***	**	0.109	0.083	–	0.313
RIAV + RIIV + RISSDV	***	0.811	***	*	**	0.466	0.313	–

**Fig. 6:**
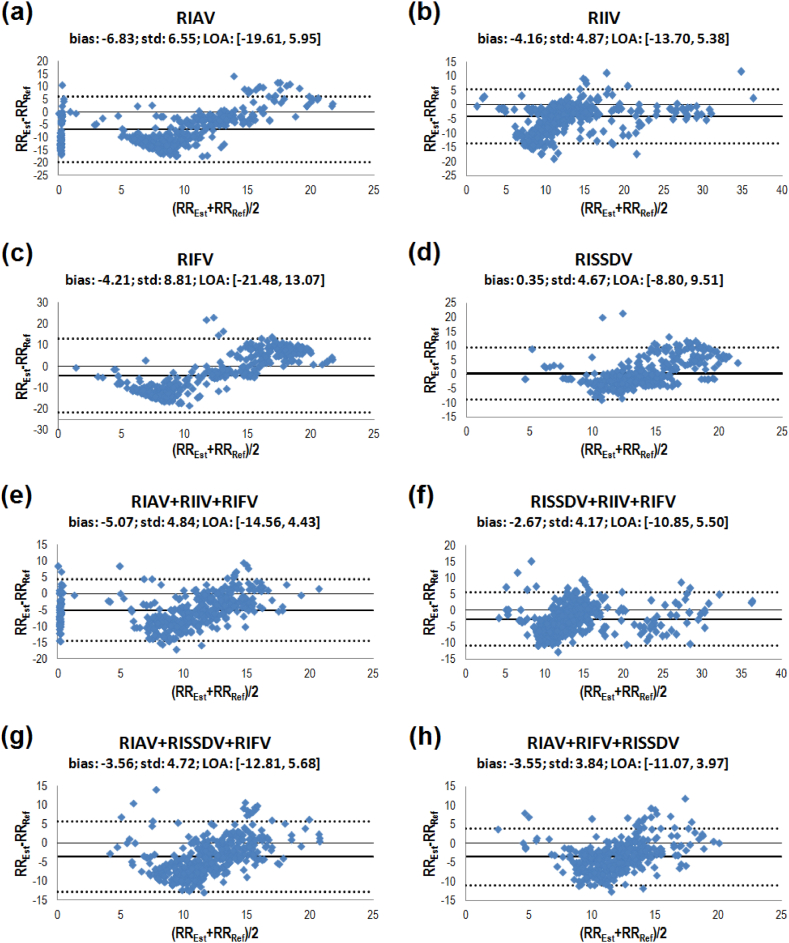
Bland-Altman plots of the PPG-derived estimations and RR reference values. The bias is shown by a bold solid line, whereas the limits of agreement (bias ± 1.96 × standard deviation) are shown by dotted lines. Panels (a–d) show the results for RR estimates based on a single RIV, while panels (e–h) illustrate those for RR estimates obtained from three-RIV combinations.

Fig. 6 illustrates the results of the Bland-Altman analysis, and Table 3 shows the mean difference's 95% confidence intervals (95% CI). RRs derived from RISSDV achieved the lowest bias and the narrowest limits of agreement (LOA). While three-RIV-based RR estimates showed similar accuracy, no single three-RIV combination simultaneously achieved the lowest bias and the narrowest LOA (see Fig. 6(e−h)). According to Table 3, only the 95% CI of the mean difference between reference and RISSDV-derived RR values contained the line of equality (i.e., *RR*_*est*_ – *RR*_*ref*_ = 0).

**Table 3:** 95% confidence intervals (CI) of the differences between PPG-derived estimations and RR reference values.

PPG-derived variation	95% CI	
	from	to
RIAV	−0.1243	−0.1034
RIIV	−0.0772	−0.0615
RIFV	−0.0843	−0.0560
**RISSDV**	**−0.0017**	**0.0133**
RIAV + RIIV + RIFV	−0.0922	−0.0767
RISSDV + RIIV + RIFV	−0.0513	−0.0379
RIAV + RISSDV + RIFV	−0.0670	−0.0518
RIAV + RIIV + RISSDV	−0.0653	−0.0530

## 4 Discussion

According to Fig. 5 and Table 1, Table 2, RMSE and MAE values shown by RISSDV were significantly lower than those of the three most widely known RIVs (i.e., RIAV, RIIV, and RIFV), as well as of some of their combinations. In the Bland-Altman analysis, RISSDV had the lowest bias, the narrower LOAs, and it was the only variation that included the line of equality (*RR*_*est*_ – *RR*_*ref*_ = 0) in the 95% confidence interval. When the line of equality is not in the confidence interval, there is a significant systematic difference between estimated and reference values [29]. Therefore, one could say that, compared with the other three RIVs, the mean difference (i.e., the bias) of RISSDV-based RR estimations is not significant, which suggests that RISSDV provides a more accurate single RIV-based RR computation from PPG signals.

**Fig. 7:**
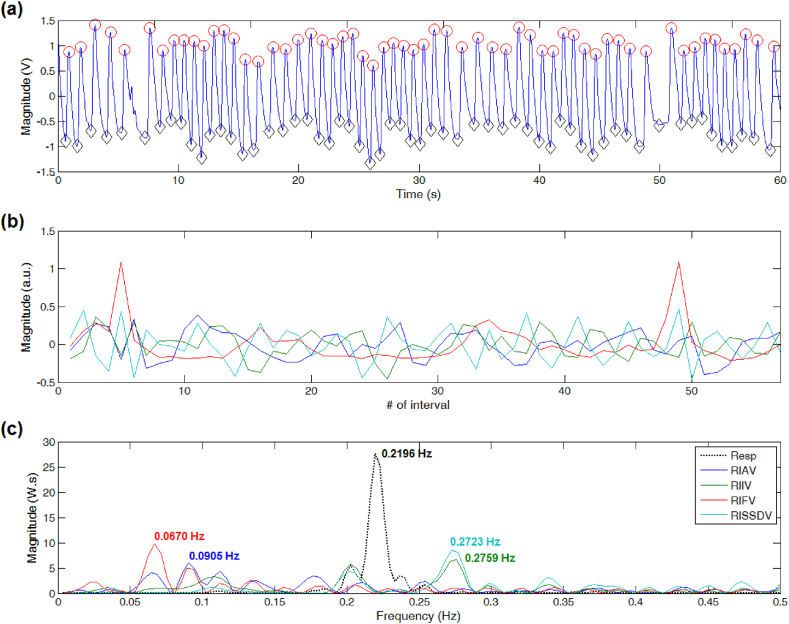
Respiratory rate (RR) estimation on a PPG signal showing irregular heart rhythm. (a) When a PPG pulse is not immediately followed by another one, the interpulse interval becomes longer than the average. (b) Respiratory-induced frequency variations are also highly sensitive to wider interpulse intervals and lose their periodicity. (c) Differences between PPG-derived RR estimations and the reference value.

Possible sources of error include PPG peak-trough detection failures, which, in turn, can be produced by abnormal or missing PPG pulses. The BIDMC records contain sections with various slightly and severely corrupted PPG signals, which can be systematically discarded to improve the accuracy of the estimated RR values. Whereas this strategy has been implemented in several studies [18,19], we analyzed each of the 53 PPG records throughout their entire length. As shown in Fig. 3, Fig. 4, the estimated RR value provided by RISSDV was closer to the reference than those of the other three RIVs for slightly and severely disturbed PPG segments, thereby suggesting that this parameter is less sensitive to failures in peak-trough detection. Some PPG recordings showed pulses that were not immediately followed by any detectable pulse, leading to longer interpulse intervals due to 'absent' or 'missing' pulses (see Fig. 7). When interpulse intervals are longer than the average, PPG frequency variations-derived respiratory signals lose their periodicity (see Fig. 4, Fig. 7), which, in turn, may hinder accurate RR estimation [30]. However, PPG amplitude variations-derived respiratory signals (e.g., RISSDV) show lower sensitivity to longer interpulse intervals, as illustrated in Fig. 4, Fig. 7. Instead, RR estimations based on amplitude variations are more sensitive to sudden signal's amplitude or baseline changes. Given that PPG recordings showing 'missed pulses' were more frequent than those containing sudden amplitude changes, the error shown by amplitude variations was considerably lower than that of estimates based on frequency variations (see Fig. 5).

Despite the above claims, the RMSE and MAE values of RIV combinations including RISSDV and RIIV (i.e., RISSDV + RIIV + RIFV and RIAV + RIIV + RISSDV) were statistically comparable with those obtained from RISSDV (see Table 1, Table 2). Thus, it is reasonable to assume that combining RISSDV and RIIV may contribute to accurate RR computation. On the other hand, several existing approaches report MAE values lower than that shown by RISSDV for the BIDMC dataset (see Table 4). Differences in estimation errors could be attributed to how many RIVs were considered for computing RR and how the information provided by each was combined. As done by several other authors [17,19], we averaged three single RIV-derived estimates to compute RR. Despite its computational efficiency, this method is strongly affected by outliers, so it is enough that one of the estimates deviates considerably from the reference value for the global error to increase. Several other methods providing lower MAE values [16,31] rely on signal decomposition schemes (e.g., empirical mode decomposition - EMD), thereby eliminating the need for detecting peaks and troughs. On the other hand, such approaches are often computationally more expensive than those based on the PPG morphology. As two of the three combinations that included RISSDV showed significantly lower errors than the well-known RIAV + RIIV + RIFV (see Table 1, Table 2), it can be concluded that combining RISSDV with other RIVs could decrease the error and improve the accuracy of average-based RR estimates. Nevertheless, it is necessary to consider different approaches and determine which and how many variations to include for computing reliable RR estimations.

**Table 4:** Comparison of the results provided by the present study with those of existing methods on the BIDMC dataset. MAE is presented as the median and inter-quartile range (25th – 75th percentiles), and all values are expressed in breaths per minute (bpm).

Author(s)	Method	MAE	Mean difference [95% CI]	Number of RIVs used for estimation
Pimentel et al. [18],	Multiple auto-regression	2.7 (1.5–5.3)	–	Three (RIAV + RIIV + RIFV)
Karlen et al. [19],	Smart fusion	5.7 (1.5–9.7)	3.0 [0.1 to 6.0]	Three (RIAV + RIIV + RIFV)
Fleming and Tarassenko [13],	Auto-regression	5.5 (2.7–8.1)	2.8 [0.7 to 4.9]	One (RIIV)
Shelley et al. [14],	Short-time Fourier transform	2.3 (0.9–7.9)	−0.4 [–2.1 to 1.3]	One (RIIV)
Nilsson et al. [32],	Regression analysis	4.6 (2.5–8.5)	1.9 [0.2 to 3.7]	One (RIIV)
Proposed	Lomb-Scargle periodogram of successive systolic differences	**3.2 (2.1**–**4.9)**	**0.4 [0.0017 to 0.0133]**	One (RISSDV)

Regarding the potential issues related to real-time implementations for calculating RR from Lomb-Scargle-based estimation of PSD, it is worth mentioning that, to the best of our knowledge, no comparison between the computational efficiency achieved by the FFT and Lomb-Scargle periodogram in estimating the PSD has been reported in the literature. According to VanderPlas [33], the Lomb–Scargle periodogram increases the number of computations needed for a problem of size N to O(N^2^) because of the involvement of trigonometric functions (it requires sums over N sinusoids). On the other hand, faster implementations have been proposed to compute the periodogram in O(N logN) time [34,35], which is the same time taken by the classical FFT. Furthermore, as the computational time scales with the number of data samples (N), the FFT will be more demanding due to the necessity of interpolating and resampling the unevenly sampled data (i.e., RIVs). In this sense, real-time applications for RR estimation from FFT-based computation of PSD will require the development of online resampling and outliers' replacement algorithms, two unnecessary tasks when the Lomb-Scargle periodogram is used [36].

The present study comes with several limitations. First, we used data acquired from hospitalized patients under stationary rather than ambulatory conditions. Therefore, the performance of RISSDV in estimating RR outside clinical settings may differ from that reported in this work. Second, the subjects' RR values are limited to the range between 5 and 25 bpm, which may preclude results generalization in a broader RR range. Third, data used for analysis were mainly from middle-aged and older adults, so pediatric and neonatal recordings are necessary for extrapolating the present findings to these populations. However, given the patients' wide age range, it would be reasonable to generalize the current results to the adult population. And fourth, we used only one segment size (1 min), and no comparison with other window lengths (e.g., 30 s) was performed. Nevertheless, our choice was based on the evidence provided by Pimentel and colleagues [18], who found that a segment or window length of 64 s can reduce MAE when used in several approaches for estimating RR, including those relying on averaging single RIV-based estimations.

## 5 Conclusion

Breathing produces well-documented changes in the amplitude, baseline, and frequency of PPG signals, so RR estimation from respiratory-induced variations in peripheral blood flow has mainly relied on these three features. On the other hand, several other parameters may also contain the information required to extract RR accurately. The present work explored the capabilities of the respiratory-induced variations in successive systolic differences (RISSDV) to estimate RR. We found that RISSDV provides significantly lower error values than well-known RIAV, RIIV, and RIFV when used for RR estimation in hospitalized adults. Moreover, RISSDV appears less sensitive to absent or missed PPG pulses and, therefore, more robust than estimations based on frequency variations. Nevertheless, further research is needed to extrapolate the present findings to subjects under non-clinical (e.g., ambulatory) conditions, including pediatric and neonatal populations.

## Ethics declarations

Review and/or approval by an ethics committee was not needed for this study because it did not include human or animal subjects and only publicly available data were used. Consent for publication is not applicable because the study did not include human subjects.

## Data availability statement

The data used in this research are publicly available at https://physionet.org/content/bidmc/1.0.0/, and the codes can be obtained under reasonable request from the corresponding author.

## Funding

This research has been funded by Dirección General de Investigaciones of 10.13039/100020560Universidad Santiago de Cali under call No. DGI-01-2024.

## Additional information

No additional information is available for this paper.

## CRediT authorship contribution statement

**Erick Javier Argüello-Prada:** Writing – review & editing, Writing – original draft, Supervision, Methodology, Formal analysis, Data curation, Conceptualization. **Katherin Daniela Marcillo Ibarra:** Writing – review & editing, Writing – original draft, Methodology, Investigation, Formal analysis, Data curation. **Kevin Leonardo Díaz Jiménez:** Writing – review & editing, Writing – original draft, Methodology, Investigation, Formal analysis, Data curation.

## Declaration of competing interest

The authors declare that they have no known competing financial interests or personal relationships that could have appeared to influence the work reported in this paper.
